# Advances in exercise snacks for interrupting sedentary behavior and promoting physical activity: a narrative review

**DOI:** 10.3389/fpubh.2026.1810516

**Published:** 2026-04-22

**Authors:** Shan Ye, Yang Ding, Beibei Hu, Na Zhou, Ruiying Huang, Yiyu Zhuang

**Affiliations:** Nursing Department, Sir Run Run Shaw Hospital, Zhejiang University School of Medicine, Hangzhou, China

**Keywords:** cardiometabolic health, exercise snacks, FITT+T, implementation, physical activity, sedentary behavior, sedentary interruption

## Abstract

Sedentary behavior is associated with higher all-cause mortality and increased cardiometabolic risk, while lack of time remains a major barrier to regular physical activity (PA). Exercise snacks are a daily-life PA strategy characterized by short, repeatable bouts of activity distributed across multiple time points throughout the day. This format may help interrupt prolonged sitting and increase overall PA exposure with minimal reliance on dedicated facilities or equipment. This narrative review, informed by a structured literature search, aimed to (i) clarify the conceptual boundaries between exercise snacks and related approaches, (ii) summarize key prescription elements—frequency, intensity, time, type, and timing (FITT+T)—and their physiological and behavioral rationale, and (iii) synthesize evidence on applications across populations and settings, associated health effects, and implementation considerations. Relevant literature was searched in PubMed (MEDLINE) and Scopus from database inception to September 30, 2025, supplemented by reference-list screening and iterative manual searches. Evidence was synthesized thematically. For health-related outcomes, systematic reviews and meta-analyses were prioritized where available, with individual randomized controlled trials and other primary studies cited selectively when needed for specific populations, outcomes, protocols, or newer evidence. Overall, current evidence suggests that exercise snacks can improve sedentary patterns and may increase activity levels in some contexts. Favorable effects have been reported for postprandial glucose and insulin responses, and some protocols sustained over several weeks have also been associated with improvements in cardiorespiratory fitness and physical function. However, evidence remains limited or inconsistent for blood lipids, body composition, psychological outcomes, and several longer-term clinical endpoints. Most studies report high acceptability and adherence, but real-world implementation may be hindered by forgetting, contextual constraints, and variability in individual capacity. Future research should establish greater consistency in terminology and FITT+T reporting, clarify dose-response relationships across outcomes and populations, leverage wearable and mobile health technologies to support detection and prompting of brief activity bouts, and conduct stratified trials with longer follow-up in high-risk and special populations to support scalable translation into public health and clinical practice.

## 1 Introduction

Sedentary behavior is typically defined as any waking behavior characterized by a sitting, reclining, or lying posture with an energy expenditure of ≤ 1.5 metabolic equivalents (METs) (1). Systematic reviews and meta-analyses have demonstrated that greater sedentary time is significantly associated with increased risks of all-cause mortality, cardiovascular disease, cancer mortality, and the development of type 2 diabetes. Each additional hour of sedentary time is linked to an approximately 2%−5% higher risk of all-cause mortality (2, 3). Accordingly, increasing physical activity (PA) and reducing sedentary behavior are central strategies for mitigating sedentary-related health threats, improving metabolic health, lowering disease risk, and enhancing quality of life (4). Health-relevant movement behavior extends beyond participation in discrete exercise sessions (5). The distribution of movement and sedentary time across the waking day also matters, particularly in environments characterized by prolonged sitting (6). Against this background, exercise snacks may offer a practical approach for embedding short activity bouts into everyday routines, thereby complementing rather than replacing traditional exercise.

The 2020 World Health Organization (WHO) Guidelines on Physical Activity and Sedentary Behavior recommend that adults accumulate at least 150–300 min of moderate-intensity or 75–150 min of vigorous-intensity aerobic activity per week, together with muscle-strengthening activities on two or more days per week (7). Importantly, these guidelines extend specific recommendations to older adults, children and adolescents, pregnant and postpartum women, and adults living with chronic conditions or disabilities, underscoring the need to consider the full spectrum of motor abilities and functional capacities, rather than aerobic capacity alone, when designing PA interventions. Conventional exercise recommendations are commonly operationalized through aerobic and muscle-strengthening activities, which differ in their primary training aims and prescription characteristics (8). Aerobic and resistance exercise are also typically performed as separate training modalities, each following distinct protocols with respect to intensity, volume, and progression (8). Although their specific effects may vary across populations and contexts, both are broadly relevant to maintaining physical function, supporting everyday activity, and promoting health (9).

However, lack of time remains one of the most frequently reported barriers to PA participation across working-age adults, caregivers, and other populations facing competing daily demands (10, 11). In real-world settings, participation in structured exercise may be constrained by scheduling demands, caregiving responsibilities, workplace routines, limited access to facilities, and the perceived need for uninterrupted exercise blocks (12). These barriers may not only reduce initiation but also undermine long-term sustainability, especially when exercise is experienced as competing with other essential daily tasks (13). Interest in exercise snacks has therefore developed alongside broader efforts to design time-efficient exercise strategies. Earlier approaches such as high-intensity interval training (HIIT), which alternates brief periods of vigorous effort with recovery intervals within a single session, and high-intensity circuit training (HICT), which combines aerobic and resistance-type stimuli in a compact format, form part of the broader evolution of time-efficient exercise prescription (14). However, unlike HIIT and HICT, which are typically organized as single structured exercise sessions, exercise snacks emphasize the distribution of brief activity bouts across multiple time points during the day, often with greater integration into daily life and greater relevance to interrupting sedentary behavior (15).

Against this broader conceptual and practical background, exercise snacks have emerged as an integrated daily-life PA strategy. By dispersing short, repeatable bouts of activity across multiple time points throughout the day, exercise snacks allow for the distributed accumulation of activity volume with minimal reliance on facilities or equipment. This approach can immediately interrupt sedentary behavior and enhance overall PA exposure (16–18). Current evidence suggests that brief bouts of moderate-to-vigorous activity may improve postprandial glucose and insulin responses in certain settings and may also promote myofibrillar protein synthesis (19). Interventions sustained over several weeks have further been associated with improvements in cardiorespiratory fitness, muscular function, and cognitive performance in some populations (20–23). Moreover, the low barrier to entry and ease of integration into daily life may reduce perceived barriers to initiation, strengthen self-efficacy, and facilitate habit formation (24). Nevertheless, important gaps remain in this field, including inconsistent terminology and prescription elements, heterogeneity in outcome assessment and evidence quality, and limited guidance on implementation strategies in real-world settings. Therefore, this narrative review aims to delineate the conceptual boundaries between exercise snacks and related approaches, summarize their prescription elements—frequency, intensity, time, type, and timing (FITT+T)—as well as their physiological and behavioral foundations, synthesize evidence on applications and health effects across diverse populations and contexts, and discuss implementation challenges and practical recommendations to support future research and translation into public health practice.

## 2 Methodology

### 2.1 Literature search and evidence identification

This study was conducted as a narrative review informed by a structured literature search. The purpose of the search was to improve transparency and breadth of coverage rather than to implement a full protocol-driven systematic review. Relevant literature was searched in PubMed (MEDLINE) and Scopus from database inception to September 30, 2025, with no restrictions on language, publication year, study design, or participant characteristics. The search strategy comprised two main concept groups. The first focused on exercise snacks and related terms, including exercise snack, exercise snacking, movement snack, Snacktivity, and vigorous intermittent lifestyle physical activity (VILPA). The second combined terms related to activity breaks or interrupting sedentary behavior with sedentary-related keywords. Full search strategies are provided in Supplementary Table S1. Records identified from both databases were merged and duplicates were removed. Study identification and selection were guided primarily by thematic relevance to the aims of the review, with particular attention to studies addressing short, repeatable activity bouts distributed across the day and studies examining interruption of sedentary behavior. Reference-list screening and iterative manual searching of relevant publications were also used to identify additional sources. Because this was a narrative review, no formal systematic-review protocol, prespecified risk-of-bias assessment, or PRISMA-style study selection process was applied. For health-related outcomes, systematic reviews and meta-analyses were prioritized where available. Individual randomized controlled trials and other primary studies were cited selectively when no relevant review addressed a specific population, outcome, or protocol; when direct citation was needed for intervention details or quantitative findings; or when a primary study postdated the most recent relevant review. No quantitative pooling across evidence types was undertaken. During manuscript revision, highly relevant publications published after the database search cutoff were identified through iterative manual updating and cited selectively.

## 3 Overview of exercise snacks

### 3.1 Concept of exercise snacks

A universally accepted definition of exercise snacks has yet to be established, and descriptions vary across the emerging literature (15, 17). In general, exercise snacks refer to short, repeatable bouts of physical activity (PA) distributed across multiple time points throughout the same day (16, 25). Individual bouts may range from a few seconds to approximately 10 min, although many protocols most commonly involve activity lasting several seconds to a few minutes (15, 25, 26). The modalities can encompass lifestyle-based activities, aerobic exercise, and resistance or bodyweight exercises, with particular emphasis on feasibility in everyday contexts and minimal reliance on dedicated facilities or equipment (15).

Exercise snacks should not be interpreted as a homogeneous physiological stimulus. Rather, the term refers primarily to a behavioral-exercise format in which brief, repeatable activity bouts are distributed across the day (22). Within this format, protocols may differ substantially in duration, intensity, structure, and likely effects. Very short bouts may be most relevant for interrupting sedentary behavior, increasing movement frequency, or contributing to accumulated activity exposure (27), whereas longer or more demanding repeated bouts may provide a greater stimulus for fitness-related or training-like adaptations. The common feature uniting this spectrum is therefore not a single duration or intensity threshold, but the temporal distribution of brief, repeatable activity bouts across multiple time points throughout the day, often embedded within everyday life (15).

From a public health and behavioral-intervention perspective, this distributed pattern is central to the appeal of exercise snacks. By accumulating activity in small increments, exercise snacks may help interrupt prolonged sedentary behavior while increasing overall PA exposure (28). At the same time, the appropriate intensity should be individualized according to population characteristics, functional capacity, health status, and intervention goals. Although some protocols involve vigorous or relatively effortful exercise, others may rely on moderate-intensity, functional, or otherwise low-barrier movement forms, particularly in older adults, sedentary individuals, or clinical populations (29). Intensity should therefore be considered an individualized prescription component rather than a defining criterion of the concept itself.

To reduce conceptual drift and improve comparability across studies, exercise snacks should also be distinguished from several adjacent concepts (Table 1). Vigorous intermittent lifestyle physical activity (VILPA) refers to brief, vigorous activity exposures that arise opportunistically during daily living, such as commuting or household tasks, without an explicit intention to exercise (30). In contrast, exercise snacks are more often planned, purposeful, and potentially anchored to fixed time points or contextual cues, making them more amenable to structured prescription as a lifestyle-integrated PA strategy (22). High-intensity interval training (HIIT) is typically organized as a single structured exercise session in which bouts of vigorous effort alternate with recovery intervals to induce training adaptations (31). Beyond the distinctions summarized in Table 1, high-intensity circuit training (HICT) similarly represents a compact session-based format combining aerobic and resistance-type demands within one continuous exercise session (14). Exercise snacks differ from these approaches in that they emphasize the distribution of brief activity bouts across multiple separate occasions throughout the day rather than within one dedicated exercise session. Accordingly, HIIT and HICT should be understood as adjacent but conceptually distinct time-efficient exercise strategies rather than interchangeable terms.

**Table 1:** Conceptual distinctions among exercise snacks, VILPA, and HIIT.

Feature dimension	Exercise snacks	VILPA	HIIT
Duration of a single bout	Seconds to ~10 min (typically seconds to a few minutes); short, repeatable bouts distributed across the day	Brief, sporadic vigorous lifestyle bouts, typically a few minutes or less	High-intensity intervals lasting several tens of seconds to a few minutes; repeated within a single session
Intensity	Moderate to vigorous; may include very high-intensity bouts (e.g., sprint-type); commonly defined by %HR, RPE, or power/speed	Vigorous-intensity daily activities; non-exercise intent and opportunistic occurrence	Vigorous-intensity, typically submaximal; commonly defined by %HR, %VO_2_ max, and RPE
Intent and structure	Planned, purposeful exercise emphasizing fragmentation and accumulation across the day; may be cue- or time-triggered	Unplanned, incidental activities occurring opportunistically; not structured exercise	Planned, structured training sessions with repeated high-intensity intervals and recovery
Frequency and intervals	Multiple times/day; inter-bout intervals from tens of minutes to several hours (≥1 h for sprint-type bouts)	Frequency and intervals determined by daily life contexts	Multiple intervals/session with recovery lasting tens of seconds to several minutes; sessions several times/week
Typical settings	Office, home, campus, stairwells, and other settings enabling integration into daily routines	Commuting, household tasks, stair climbing, and other everyday life activities	Gyms, laboratories, sports facilities, or adapted home-based settings

This table summarizes key conceptual distinctions among exercise snacks (15), vigorous intermittent lifestyle physical activity (VILPA) (30), and high-intensity interval training (HIIT) (31). Typical settings are illustrative examples and do not constitute defining criteria.

In Chinese health communication, the term “fragmented exercise” is sometimes used as a lay description of short, accumulated activity performed intermittently across the day. However, for academic discussion and evidence synthesis, this review consistently adopts the term “exercise snacks” in order to minimize interpretive bias arising from inconsistent terminology (32).

### 3.2 Intervention prescription using the FITT+T framework

Most exercise snacks interventions can be described using the Frequency, Intensity, Time, Type, and Timing (FITT+T) framework (Table 2). The FITT+T framework is a general exercise prescription structure and is applied here pragmatically to support the clear description and comparison of brief, distributed activity interventions (33). Importantly, Table 2 presents representative examples reported in the literature and should not be interpreted as recommended, minimum effective, or universally appropriate doses.

(1) **Frequency:** Exercise snacks are typically performed multiple times per day, although the reported frequency varies substantially across studies. Representative protocols have included, for example, 2–4 bouts/day, whereas other interventions prescribe more frequent interruptions of sedentary time or repeated activity linked to daily events (29, 34). Such values should be interpreted descriptively rather than prescriptively. Whether a given frequency is sufficient depends on the target outcome, the intensity and duration of each bout, the accumulated daily and weekly dose, the intervention duration, and the characteristics of the population studied (30). Exercise snacks should therefore not be assumed to replace structured exercise programs designed to improve cardiorespiratory fitness or develop broader motor capacities; rather, they are better understood as a complementary strategy for increasing daily PA and interrupting sedentary behavior (18).
(2) **Intensity:** Exercise snacks may range from moderate-intensity movement to vigorous or relatively effortful exercise, and in some protocols may be performed to volitional fatigue (21). However, several studies have not reported quantitative measures such as HR or RPE, relying instead on instruction-only descriptions. This limitation may contribute to heterogeneity in outcomes and uncertainty in dose-response interpretations.
(3) **Time:** Individual bouts may range from only a few seconds to several minutes, with many protocols falling within the range of several seconds to a few minutes (17, 18). However, the relevance of bout duration should not be interpreted in isolation. The likely effects of a given protocol depend on the interaction among time per bout, frequency, intensity, total accumulated dose, and intervention duration (16). Accordingly, exercise snacks should be understood as a distributed exercise stimulus rather than evaluated solely by the duration of any single bout.
(4) **Type:** Exercise snacks encompass a wide range of modalities, including aerobic activities such as stair climbing or brisk walking; resistance or bodyweight exercises such as squats, calf raises, or push-ups; mixed routines; and movement-based activity bouts such as Tai Chi (19, 35–37). However, this categorization is primarily pragmatic, as most real-world activities involve hybrid demands across multiple physiological and motor domains. For example, stair climbing and repeated sit-to-stand actions may simultaneously involve cardiorespiratory demand, strength-endurance, coordination, and balance (38). Therefore, activity “type” should not be interpreted as corresponding exclusively to a single motor ability, but rather as a dominant training characteristic within a broader multi-component effort.
(5) **Timing/triggering:** Timing is a particularly important dimension of exercise snacks because it reflects how activity is distributed across the day. One strategy involves interrupting sedentary behavior at fixed intervals, such as every 30 or 60 min (interval-based) (34, 36). Another links exercise snacks to specific events or contextual cues, such as before meals, between tasks, after toileting, or during commuting transitions (event-based/context-linked) (23, 28). These triggering structures are relevant not only to dose accumulation, but also to feasibility, adherence, and habit formation in real-world settings.

**Table 2:** Summary of exercise snack interventions across different populations.

Population (setting)	Dose (frequency × time)	Intensity	Type	Timing/ triggering	Duration	Key outcomes	**Ref**.
Sedentary young adults (stairwell)	3 times/day × ~20–22 s/bout (3 days/week)	as fast as possible (RPE ~5)	Stair ascent	≥1 h apart	6 weeks	Improved VO_2_ peak and Wpeak	(21)
Healthy older adults (home)	2 times/day × ~9 min/bout	Bodyweight AMRAP	Functional leg circuit (incl. STS)	Scheduled (AM/PM)	28 days	Improved 60-s STS performance	(22)
Adults with insulin resistance (lab/clinical)	3 times/day × 6 × 1 min + 1-min recovery (~12 min/session)	90% HRmax	Incline treadmill intervals	30 min pre-meal	5-day crossover	Reduced postprandial and 24-h glucose	(28)
Sedentary university workers (workplace)	4–16 times/day × 3 min/break (progressed)	NR	Resistance breaks	App prompts	2 × 5-day crossover	Acceptability highest at ~4/day; improved sit-to-stand transitions/stepping	(36)
Children, early puberty (school)	multiple times/day × ~3 min/day	NR	Jumping (impact)	School bell/recess	8 months	Improved femur BMC	(48)
Adolescents with type 1 diabetes (home)	3 times/day × 6 × 1 min/session (18 min/day)	OMNI resistance exercise scale ~3; progressive resistance bands	Resistance (bands)	30 min pre-meal	3 months	Reduced body fat; glycemia unchanged	(50)
Pre-frail older adults (home)	2 times/day × ~9 min/snack	progressive (graded)	Strength plus Tai Chi snacks	Scheduled daily	12 weeks	Improved balance and mobility	(54)
Pre-frail + MCI (memory clinic; home)	2 times/day × ~9 min/snack	AMRAP; RPE recorded	Strength circuit	Scheduled (flexible)	28 days	Improved SPPB; reduced TUG; adherence ~85%	(55)
Sedentary obese adults (stair protocol)	6 times/day × 4 flights (60 steps; ~23 s) (4 days/week)	fast but safe; challenging pace	Stair climbing	>1 h apart	12 weeks	Reduced visceral fat and epicardial fat	(58)

AMRAP, as many rounds as possible; BMC, bone mineral content; HRmax, maximal heart rate; MCI, mild cognitive impairment; OMNI-RES, OMNI resistance exercise scale; RPE, rating of perceived exertion; SPPB, Short Physical Performance Battery; STS, sit-to-stand; TUG, Timed Up and Go; VO_2_peak, peak oxygen uptake; NR, not reported.

Overall, substantial variability remains across studies in how FITT+T elements are defined, measured, and reported. This heterogeneity limits cross-study comparability and complicates interpretation of dose-response relationships. Future research should therefore establish minimum FITT+T reporting standards, clearly distinguish sedentary interruption-based protocols from distributed exercise-based protocols, and improve the quantification and reporting of exercise intensity and accumulated dose to strengthen both interpretability and translational potential.

### 3.3 Theoretical foundation of exercise snacks

The development and dissemination of exercise snacks are underpinned by both physiological and behavioral science theories. Physiological perspectives provide mechanistic explanations for their potential health benefits, whereas behavioral frameworks guide real-world implementation, adherence, and long-term maintenance.

#### 3.3.1 Physiological theory

The physiological relevance of exercise snacks should be interpreted according to the target outcome rather than assumed to be uniform across all domains. From a classical exercise physiology perspective, very brief efforts are less likely to induce the same adaptations as longer continuous exercise when considered in isolation (39). Accordingly, exercise snacks should not be interpreted as a homogeneous training stimulus. Their expected effects depend on the interaction among bout duration, intensity, frequency, accumulated daily or weekly dose, intervention duration, and participant characteristics (22). An important conceptual basis for exercise snacks is the cumulative nature of physical activity and the dose–response relationship between movement exposure and health (15). The 2020 World Health Organization guidelines no longer require physical activity to be accumulated in bouts of at least 10 min, emphasizing instead that physical activity of any duration can contribute to total volume (7). However, this principle should not be taken to imply that all bout durations are physiologically equivalent. Rather, it supports the view that brief activity bouts may still be meaningful, particularly when accumulated across the day and interpreted in relation to specific outcomes.

At the level of acute responses, very brief or short bouts may be especially relevant for interrupting prolonged sedentary physiology. Activity interruptions have been associated in some settings with improved postprandial glucose and insulin responses, likely in part through contraction-mediated glucose uptake (28), and with favorable acute vascular responses related to lower-limb blood flow and shear stress patterns (35, 40). These effects are particularly relevant to the interruption of prolonged sitting and to short-term metabolic regulation, rather than necessarily to traditional endurance or strength adaptations. At the level of chronic adaptations, however, improvements in outcomes such as cardiorespiratory fitness, muscular performance, or body composition are more likely to depend on sufficient intensity, repeated exposure over time, and adequate accumulated dose rather than on bout duration alone (20). More demanding or repeated protocols implemented over several weeks may provide a training-like stimulus capable of improving selected fitness-related outcomes in some populations (20–23). By contrast, very low-dose or very brief protocols should not be assumed to produce equivalent effects across all physiological or motor domains. Thus, representative frequencies reported in the literature, such as 2–4 bouts/day, should be interpreted descriptively rather than prescriptively, and their likely effectiveness should be matched to the outcome under consideration.

Taken together, these considerations suggest that exercise snacks are best understood as a distributed exercise stimulus rather than judged solely by the duration of any single bout (15). Their potential value lies not only in the isolated effect of one brief activity exposure, but also in the accumulated and temporally dispersed pattern through which activity is embedded across the waking day. Future research should further clarify dose-response relationships and determine which combinations of frequency, intensity, time, and intervention duration are required to elicit meaningful effects on different outcomes across populations.

#### 3.3.2 Behavioral science theory

The uptake and maintenance of exercise snacks can be interpreted and optimized through behavior change theories, particularly the COM-B model, self-determination theory (SDT), and habit formation theory. **(1) The COM-B model** offers a systematic framework for understanding exercise behavior change. In terms of Capability, the brief duration, flexible activity options, and scalable intensity of exercise snacks reduce fitness and skill barriers. Repeated successful completion may also provide mastery experiences that strengthen self-efficacy (37). Regarding Opportunity, minimal dependence on facilities or equipment facilitates integration into home and workplace routines, thereby expanding feasible opportunities for participation. With respect to Motivation, low barriers to initiation and an immediate sense of accomplishment may reduce psychological resistance. In addition, reminders, feedback, and tracking functions provided by wearable devices or mobile applications can enhance self-monitoring and sustained engagement (41). **(2) Self-determination theory** highlights autonomy, competence, and relatedness as essential psychological needs for maintaining health behaviors (42). Exercise snacks allow individuals substantial autonomy in timing, location, and activity type. Graded prescriptions and progressive challenges can support competence, while peer- or team-based activities and community interaction may enhance feelings of social connection and support. **(3) Habit formation theory** emphasizes the “cue–routine–reward” loop as a pathway to behavioral automaticity (43). Exercise snacks can be anchored to stable contextual cues (e.g., taking the stairs during commuting or standing up when getting water), establishing clear triggering rules. When actions and timing remain relatively consistent, repeated practice combined with immediate reinforcement—such as subjective improvements in energy levels or digital feedback—may increase the likelihood of long-term maintenance. Overall, these theoretical perspectives collectively suggest that the successful implementation of exercise snacks depends not only on what activities are performed, but also on when they are prompted, how they are scaled, how feedback is delivered, and how social support is leveraged. This provides an actionable theoretical foundation for future prescription development and implementation strategies.

## 4 Applications of exercise snacks for interrupting sedentary behavior and promoting physical activity

### 4.1 Applications in children and adolescents

Childhood and adolescence are important not only for the establishment of lifelong physical activity (PA) habits, but also for the development of motor coordination, movement skills, and selected physical capacities. These life stages include developmentally sensitive periods during which the neuromuscular and musculoskeletal systems may be especially responsive to appropriate movement exposure (44). In this context, the potential value of exercise snacks in young people lies not only in increasing activity volume or interrupting sedentary behavior, but also in providing age-appropriate and varied movement experiences that may support motor development and movement confidence (45).

Sedentary behavior is highly prevalent in this population, particularly in the context of increasing academic demands and greater exposure to screen-based activities (46). Time windows such as school recess, breaks between classes, transitions during the school day, and pauses during home study may therefore provide practical contexts for implementation (44). In later childhood and adolescence, protocols may progressively incorporate more structured, task-oriented, or skill-focused activities, depending on developmental stage, setting, and intervention goals (47). Because maturation-related changes during adolescence can also influence coordination, movement control, and physical performance, exercise snacks for young people should be developmentally tailored rather than designed simply as scaled-down versions of adult protocols. Existing interventions illustrate the diversity of possible applications. For example, McKay et al. (48) implemented the “Bounce at the Bell” program at scheduled times during the school day, with children performing 10 countermovement jumps three times per day (total <3 min/day). After 8 months, early pubertal children exhibited 2.0% and 2.7% greater increases in bone mineral content at the proximal femur and intertrochanteric region, respectively, compared with controls, suggesting potential benefits for bone health. Evidence regarding metabolic and behavioral outcomes is also gradually emerging. Ajibewa et al. (49) reported that interrupting sedentary behavior with higher-intensity activity did not induce compensatory eating. Hasan et al. (50) found that among adolescents with type 1 diabetes, a 3-month intervention involving resistance-type exercise snacks performed three times daily was associated with a reduction in body fat of approximately 2.2 percentage points.

Importantly, the available literature remains heterogeneous with respect to outcomes, populations, and prescription characteristics. Existing studies have examined diverse targets, including bone health, appetite regulation, body composition, and movement-related outcomes, and findings should therefore not be generalized across age groups or developmental stages without caution. From a practical perspective, age-appropriate design is essential. Peer interaction, playful competition, and enjoyable task variation may enhance engagement and adherence, whereas higher-impact activities such as jumping require appropriate technical instruction and supervision to reduce injury risk. Coordinated implementation across school and family settings may further strengthen sustainability. Overall, future high-quality studies should clarify which types, doses, and timing patterns of exercise snacks are most appropriate for different developmental stages and should evaluate longer-term effects across broader educational and community settings.

### 4.2 Applications in healthy adults

Healthy adults, particularly sedentary office workers, often face prolonged periods of static work alongside substantial time constraints (11). Exercise snacks can be readily integrated into everyday micro-contexts, such as workstations, break rooms, home offices, and stairwells encountered during commuting, making this approach highly practical. Current prescriptions generally fall into two broad categories: ① interrupting sedentary behavior at fixed intervals, and ② distributing higher-intensity or effortful activity bouts across multiple occasions throughout the day. Representative protocols include stair-climbing sprints performed three times per day on 3 days per week (approximately 20 s per bout, climbing three flights of stairs) (21); completing 15 bodyweight squats or 2 min of walking every 30 min (36); and using mobile applications or smartwatches to deliver scheduled or context-sensitive prompts and feedback (41). Jenkins et al. (21) reported that stair-climbing sprint protocols were associated with increases in VO_2_ peak and peak power output. Resistance-type exercise snacks may also confer meaningful benefits. Among female office employees with limited engagement in resistance training, a 12-week intervention consisting of 10 min per day, 5 days per week was associated with an increase in muscle mass (approximately 0.42 kg) and trends toward improvements in strength in selected muscle groups (51). However, these effect sizes are typically derived from individual studies, and differences in prescription elements, outcome measures (e.g., VO_2_ peak), and participant characteristics warrant caution when making cross-study comparisons or generalizations. From an implementation standpoint, anchoring exercise snacks to repeatable work or daily-life events may enhance consistency. Providing graded intensity options and alternative movements is also important to accommodate variations in fitness levels and spatial limitations. At the organizational level, establishing relatively private, non-disruptive micro-spaces and fostering team-based challenges or peer participation may reduce perceived stigma and psychological barriers. Finally, wearable technologies can support individualized reminders and motivational reinforcement, thereby facilitating sustained engagement (41).

### 4.3 Applications in older adults

Older adults commonly experience multiple age-related challenges, including declines in muscle mass and strength, impaired balance, progression of frailty, and cognitive impairment (52). Accordingly, the promotion of exercise snacks in this population should prioritize safety, accessibility, and low barriers to participation. Implementation is therefore often centered on home-based settings and long-term care facilities (53). Emerging evidence suggests that short, repeatable practices focused on functional tasks can be tailored to different risk profiles and outcome targets. With respect to muscular function, Perkin et al. (22) implemented sit-to-stand exercises twice daily in healthy older adults. After 28 days, 60-s sit-to-stand performance improved by 31%, maximal leg press power increased by 6%, and thigh muscle cross-sectional area increased by 2%. In terms of balance and mobility, Liang et al. (54) reported that a 12-week Tai Chi–based exercise snacks intervention improved SPPB balance scores by 0.65 points. Furthermore, in a lab-based subset, total SPPB scores improved by 1.76 points. Among prefrail individuals with mild cognitive impairment attending memory clinics, Western et al. (55) found that 28 days of twice-daily muscle-strengthening exercise snacks increased the median Short Physical Performance Battery (SPPB) score from 8 to 9 and reduced Timed Up and Go completion time by approximately 2.1 s, with reported adherence of ~85% among participants who attempted the full intervention. Importantly, these findings originate from heterogeneous populations and prescription protocols, and the magnitude and generalizability of effects require confirmation in larger samples with longer follow-up. From an implementation perspective, healthcare providers should assess cardiovascular risk, fall risk, balance function, and cognitive status prior to intervention. Clear movement demonstrations should be provided, and caregiver supervision may be necessary for individuals at higher risk. Mobile health technologies can assist with sedentary interruption prompts and ongoing feedback (56); however, the digital divide among older adults must be addressed through simplified user interfaces, targeted training, and support from caregivers to ensure accessibility and sustained engagement.

### 4.4 Applications in patients with chronic diseases

Patients with chronic diseases often experience reduced physical capacity, multimorbidity, and complex medication regimens. Accordingly, the implementation of exercise snacks in this population should adhere to principles of medical supervision and individualized prescription. Relevant settings primarily include home-based environments as well as clinical and community rehabilitation contexts. Among individuals with insulin resistance, one study implemented uphill treadmill brisk walking before each of the three daily meals (6 bouts × 1 min, interspersed with 1 min of slow walking recovery; intensity reaching approximately 90% of maximal heart rate (HRmax), yielding ~18 min of high-intensity exercise per day). Compared with traditional continuous moderate-intensity exercise, this protocol attenuated 3-h postprandial glucose (notably after breakfast and more than continuous exercise after dinner) and reduced 24-h mean glucose, with reductions persisting for the subsequent 24 h (28). These findings suggest potential utility in postprandial glycemic management. Rafiei et al. (57) interrupted 9 h of sitting with hourly stair-climbing snacks consisting of ascending three flights (55 steps) in 15–30 s, performed eight times across the day. In adults with overweight/obesity, this reduced 9-h insulin AUC by 16.5% and NEFA AUC by 21% vs. uninterrupted sitting. Furthermore, a randomized controlled trial reported that a 12-week stair-climbing exercise snacks intervention (4 days/week, 6 bouts/day, four flights totaling 60 steps per bout completed within 20–30 s, with intervals ≥1 h) was associated with reductions in abdominal visceral fat and epicardial adipose tissue (58). Regarding blood pressure management, accumulated exercise appears to confer antihypertensive effects broadly comparable to those of a single continuous bout, with some evidence indicating greater improvements in diastolic blood pressure. For instance, dividing 40 min of continuous brisk walking into four daily bouts of 10 min produced similar reductions in systolic blood pressure and more favorable effects on diastolic blood pressure (59). Importantly, these prescriptions have largely been derived from specific research settings and relatively selected samples, with differing requirements for facilities, baseline fitness, and risk management. Therefore, real-world implementation should avoid uncritical replication. In practice, cardiovascular risk assessment and screening for exercise contraindications should be conducted prior to initiation. Prescriptions should be graded and dynamically adjusted according to disease type, severity, and baseline functional capacity. Patients with diabetes should monitor blood glucose before and after exercise and establish hypoglycemia prevention protocols. Patients with cardiovascular disease may require heart rate and blood pressure monitoring, clearly defined stopping thresholds, and procedures for responding to warning symptoms. Finally, family support, peer communities, and appropriate psychological support may enhance motivation and promote long-term adherence.

## 5 Effectiveness of exercise snacks

### 5.1 Behavioral outcomes

Evidence on behavioral outcomes has primarily been derived from workplace- and home-based intervention studies. Commonly reported indicators include the frequency of sit-to-stand transitions, the longest uninterrupted sedentary bout, total sedentary time, as well as step counts and time spent in moderate-to-vigorous physical activity (MVPA). Overall, the behavioral impact of exercise snacks is mainly reflected in two domains: improving sedentary patterns by interrupting prolonged sitting and increasing overall physical activity exposure. Interrupting sedentary behavior is achieved by segmenting extended sitting periods through frequent, brief bouts of activity. Rogers et al. (36) demonstrated that resistance-type exercise snacks significantly increased sit-to-stand transitions among office employees. Several interventions have adopted a strategy of breaking up sedentary time every 30 min (19, 36), thereby limiting the longest continuous sedentary bout to ≤ 30 min. Such changes may improve sedentary continuity, a key risk-related characteristic of sedentary behavior. In addition, exercise snacks may enhance overall activity exposure by leveraging the accumulation of physical activity in small increments, thereby providing a practical pathway toward meeting public health guideline targets. Snacktivity™ encourages multiple activity bouts of 2–5 min throughout the day (24, 60), making it easier to accumulate weekly MVPA toward the recommended 150 min. Maylor et al. (61) reported that integrating exercise snacks into home and workplace routines was associated with a net increase of approximately 4,500 steps per day, an increase in MVPA of 20.3 ± 2.1 min, and an increase in daily energy expenditure of 661 kJ. Notably, some studies indicate that while exercise snacks can effectively interrupt sedentary patterns, reductions in total sedentary time are not consistently observed (50). This may be because interventions fragment long sedentary bouts into shorter ones without decreasing overall sedentary time, because compensatory sedentary behavior occurs outside intervention periods, or because the prescribed intensity or dose is insufficient to drive meaningful reductions in sedentary duration. These findings suggest that behavioral outcomes may depend on the specific intervention targets and the extent to which compensatory behaviors are addressed. Overall, evidence for improvements in sedentary bout patterns appears more consistent, whereas evidence for reducing total sedentary time remains less robust. Further optimization of prescription dose and contextual embedding strategies is warranted.

### 5.2 Clinical outcomes

Exercise snacks have demonstrated promising but heterogeneous effects across a range of clinical outcomes. Review-level evidence suggests that exercise snacks may improve selected cardiometabolic and fitness-related outcomes in adults, whereas evidence for consistent benefits in body composition, blood pressure, blood lipids, and several other health indicators remains limited or inconsistent (15, 20). Acute metabolic benefits appear to be more evident in specific populations and intervention contexts. For example, in individuals with insulin resistance, pre-meal exercise snacks have been associated with attenuated postprandial glucose responses and lower 24-h mean glucose (28). Similarly, in adults with overweight or obesity, interrupting prolonged sitting with stair-climbing exercise snacks reduced postprandial insulin exposure, although effects on glucose were less clear (57). A randomized controlled trial also reported reductions in abdominal visceral fat and epicardial adipose tissue following a 12-week stair-climbing exercise snacks intervention in sedentary adults with obesity (58), although such findings have not yet translated into consistent review-level support for body-composition benefits overall (20). In musculoskeletal health, current evidence also remains heterogeneous. Review-level evidence indicates potential benefits for physical function and muscular outcomes in older adults, but the number of trials remains limited and intervention protocols differ substantially (15). Therefore, individual primary studies were retained only to illustrate representative intervention models, such as sit-to-stand–based exercise snacks, Tai Chi–based exercise snacking, and remotely delivered resistance-oriented protocols in older adults (22, 26, 54). Psychological and cognitive outcomes remain less conclusive. Although some studies suggest improvements in perceived energy, mood, or aspects of executive function, the overall evidence is mixed and effect sizes appear modest (15, 23). Taken together, current evidence supports the potential of exercise snacks for improving selected metabolic, fitness, and physical function outcomes, but more adequately powered studies with standardized prescriptions and longer follow-up are needed to clarify dose–response relationships and population-specific effects.

### 5.3 Adherence, feasibility and safety

Review-level evidence indicates that exercise snacks are generally feasible, acceptable, and safe in adults and older adults when implemented in home-, community-, or workplace-based settings (15). High adherence has been reported across many studies, likely reflecting the flexibility, time efficiency, and minimal equipment requirements of this approach (15). However, feasibility appears to vary according to context, delivery mode, and population characteristics. Primary qualitative and mixed-methods studies suggest that participants may forget to complete repeated daily bouts, encounter scheduling conflicts during busy periods, or require additional prompting and support to sustain engagement over time (29, 37, 62). These findings indicate that reminder systems, contextual cues, and individualized support strategies may be important for real-world implementation.

In terms of safety, serious adverse events have rarely been reported in the existing literature, but adverse event monitoring and reporting remain inconsistent across studies (15). In addition, most available studies are short-term and involve relatively small samples, which limits inference regarding rare adverse outcomes and long-term safety in higher-risk populations. Representative primary studies in older adults have also reported favorable acceptability and no major safety concerns under supervised or remotely supported conditions (26, 37), although these findings should be interpreted with caution because of limited sample sizes and short intervention durations. Accordingly, future research should strengthen adverse event surveillance, incorporate longer follow-up, and establish clearer safety guidance for older adults, individuals with functional limitations, and patients with chronic diseases.

## 6 Current challenges and future directions

Although exercise snacks have demonstrated promising potential for reducing sedentary behavior, increasing physical activity exposure, and improving selected metabolic and fitness indicators (15, 16, 20), real-world implementation and scale-up remain constrained by challenges related to feasibility, comparability, and sustainability. Future research should adopt population- and context-centered approaches, while concurrently optimizing implementation strategies, standardizing prescription and reporting practices, advancing monitoring technologies, and strengthening interdisciplinary collaboration. These efforts are critical to translate exercise snacks from proof-of-concept into replicable public health and nursing practice.

### 6.1 Real-world implementation and user-centered challenges

Exercise snacks are often described as flexible and easy to integrate into daily life, but they are not universally easy to adopt or sustain across all settings. Their real-world appeal lies partly in the fact that they may reduce some of the structural demands associated with conventional exercise, such as the need for dedicated time blocks, specific facilities, or uninterrupted sessions (22). However, the same daily contexts in which exercise snacks are intended to be embedded may also create competing demands that limit uptake and long-term maintenance.

In home, workplace, and community settings, participation may be constrained by scheduling demands, caregiving responsibilities, workplace routines, limited private space, competing task priorities, and the perceived impracticality of interrupting ongoing activities for repeated movement breaks (11). Qualitative studies further suggest that participants may forget to engage in exercise snacks, find the timing burdensome during busy periods, or experience difficulty integrating repeated bouts into socially or professionally structured routines (29, 37). These barriers are important because they indicate that low theoretical burden does not automatically translate into sustained real-world adherence.

Accordingly, successful implementation is likely to depend not only on the exercise content itself, but also on how the activity is prompted, timed, and embedded within everyday routines. Interventions should therefore anchor exercise snacks to frequent and meaningful contextual cues, such as meals, work transitions, toileting, commuting, or other stable daily events, with clearly specified triggering rules. This cue-based approach may reduce reliance on continuous self-regulation and better support repetition across the waking day.

User-centered tailoring is also essential. Graded activity options, alternative movement choices, and clear safety guidance are needed to accommodate differences in functional capacity, motivation, health status, and environmental constraints, particularly among older adults and individuals with chronic diseases. In some cases, lower-intensity or function-oriented activities may be more acceptable and sustainable than more effortful protocols. Supportive physical and social environments within households, workplaces, schools, and care settings may further enhance uptake by reducing embarrassment, increasing perceived legitimacy, and facilitating shared participation.

Overall, real-world implementation should not be understood simply as a matter of prescribing short bouts of activity. Rather, it requires attention to behavioral cueing, contextual fit, feasibility, and sustained acceptability. Future implementation research should therefore examine which combinations of timing strategies, prompting systems, environmental supports, and individualized adaptation are most effective for maintaining exercise snacks across different populations and settings.

### 6.2 Urgent need for standardization of exercise prescription and reporting

Marked heterogeneity persists in terminology and operational definitions, with concepts such as exercise snacks, Snacktivity™, and VILPA often used in parallel. In addition, FITT+T elements are inconsistently defined, measured, and reported, limiting cross-study comparability and robust evidence synthesis (15, 17). Expert consensus processes are therefore needed to establish core definitions and minimum reporting standards. At a minimum, reporting should standardize bout duration ranges, intensity criteria (e.g., heart rate, RPE, power output), triggering mechanisms, and methods for calculating total dose. Studies should also clearly distinguish between “sedentary interruption—based” protocols (fixed intervals) and “distributed exercise—based” protocols (multiple daily occasions). Finally, evidence mapping frameworks may help identify effective intervention ranges tailored to specific populations and contexts.

### 6.3 Monitoring and digital health technologies: from detection to optimization

In real-world settings, very brief and high-frequency activity bouts place greater demands on detection algorithms used in commercial wearable devices. At the same time, mobile health (mHealth) tools provide a feasible pathway for establishing a closed-loop system of “prompt–action–feedback” (28). Future work should prioritize the development and validation of algorithms capable of accurately identifying short, high-intensity bouts under ecologically valid conditions. mHealth-based individualized prompts and feedback may reduce forgetting and the burden of execution. Importantly, the digital divide among older adults must be addressed through simplified user interfaces, targeted training, and nursing or caregiver support to enhance accessibility and sustained use.

### 6.4 Limited evidence in vulnerable and high-risk populations: the need for stratified and multi-setting validation

Although existing evidence has included children, adults, older adults, and certain chronic disease populations, high-risk groups—such as hospitalized patients, functionally impaired older adults, and individuals with severe chronic conditions or multimorbidity—remain underrepresented in high-quality research. Future work should prioritize multicenter, stratified randomized trials to determine safe and effective intervention doses and to establish risk management pathways across varying disease states and functional levels. Standardized, actionable tools for risk assessment and graded prescription should be developed and validated in both home-based and clinical rehabilitation settings. Moreover, scalable implementation strategies should be explored in long-term care institutions and other high-need contexts (53).

### 6.5 Limitations and future research directions

Several limitations in the current evidence base should be acknowledged. First, the literature on exercise snacks remains heterogeneous in terminology, intervention design, participant characteristics, and outcome selection. Across studies, exercise snacks have been operationalized using different combinations of frequency, duration, intensity, exercise modality, and timing, making direct comparison difficult and limiting the strength of cross-study inferences. This heterogeneity also means that exercise snacks should not be interpreted as a single, uniform intervention with predictable effects across all populations and outcomes. Second, much of the available evidence is derived from relatively small trials, short intervention periods, and selected populations, which may limit statistical power, external validity, and generalizability. In addition, several studies have focused on acute responses or highly specific endpoints, whereas longer-term adaptations in broader physiological, behavioral, and clinical domains remain less well characterized. The evidence base is therefore still insufficient to determine whether findings observed under controlled conditions can be consistently reproduced across real-world settings. Third, the relationship between exercise snack prescription and outcome response remains insufficiently understood. For example, protocols that are sufficient to interrupt sedentary behavior or improve acute glycemic responses may not necessarily provide a large enough stimulus to induce meaningful changes in cardiorespiratory fitness, muscular strength, or body composition. Future research should therefore clarify the dose-response relationship of exercise snacks by identifying which combinations of frequency, duration, intensity, accumulated volume, and timing are required for different outcomes and populations, rather than assuming that all forms of brief intermittent activity confer the same benefits. Fourth, methodological inconsistencies continue to constrain interpretation. Greater standardization is needed in the reporting of intervention characteristics, adherence, progression, contextual delivery, and comparator conditions. Future studies would benefit from clearer descriptions of whether exercise snacks are interval-based, event-based, or context-triggered, as well as from more consistent reporting of exercise intensity relative to individual capacity. Improved harmonization of outcome measures would also strengthen evidence synthesis and facilitate more meaningful meta-analytic comparisons. Finally, future work should extend beyond efficacy and address implementation more directly. Long-term adherence, acceptability, contextual fit, and feasibility across homes, schools, workplaces, and clinical settings require further investigation. Research should also examine how prompting strategies, environmental supports, user-centered tailoring, and digital tools may influence sustained uptake. Taken together, future high-quality studies should combine stronger methodological rigor with greater attention to real-world implementation in order to determine when, for whom, and under what conditions exercise snacks are most effective.

## 7 Conclusion

Exercise snacks represent a flexible and potentially scalable approach to promoting physical activity by embedding short bouts of movement within everyday life. Rather than functioning as a replacement for traditional structured exercise, they may serve as a complementary strategy that is particularly relevant for individuals who face practical barriers to longer, continuous exercise sessions. Their value appears to lie not only in increasing opportunities for movement, but also in interrupting sedentary behavior and offering a more context-embedded, potentially lower-burden format for activity accumulation across the day. Current evidence suggests that exercise snacks can improve sedentary patterns and may confer benefits across selected physiological, behavioral, and clinical outcomes. However, the literature remains heterogeneous with respect to terminology, intervention characteristics, populations, and measured endpoints. Accordingly, exercise snacks should not be regarded as a single, uniform intervention, nor should all brief activity bouts be assumed to produce the same adaptations. Their effects are more likely to be outcome-specific and dose-dependent, with the magnitude and nature of benefits influenced by factors such as exercise modality, frequency, duration, intensity, accumulated volume, timing, and population characteristics. From an applied perspective, the promise of exercise snacks will depend not only on efficacy under controlled conditions, but also on feasibility, acceptability, and contextual fit in real-world settings. Effective implementation is therefore likely to require developmentally and clinically appropriate tailoring, meaningful integration into daily routines, and user-centered design that supports sustained participation across diverse populations and environments. Overall, exercise snacks are a promising addition to the broader physical activity promotion toolkit. Future high-quality research should clarify optimal prescription characteristics, strengthen methodological consistency, and determine for whom, in which settings, and for which outcomes exercise snacks are most effective.
