# Supplementary material for: Advances in exercise snacks for interrupting sedentary behavior and promoting physical activity: a narrative review
Source: Front Public Health. 2026 Apr 22;14:1810516. doi: 10.3389/fpubh.2026.1810516 (PMC13144037; doi:10.3389/fpubh.2026.1810516)
Supplement: Supplementary file 1 [file Table_1.docx]

**Table S1 Detailed search strategies and results**

| **Database**  **(Date of Search)** | **Search Query / Syntax** | **Limits/ Filters** | **Results**  **(n)** |
| --- | --- | --- | --- |
| PubMed (MEDLINE) (30 Sep 2025) | ( "exercise snack*"[tiab] OR "exercise snacking"[tiab]  OR "movement snack*"[tiab] OR snacktivity[tiab]  OR snacktivity*[tiab] OR VILPA[tiab] OR "vigorous intermittent lifestyle physical activity"[tiab] OR "intermittent lifestyle physical activity"[tiab]) OR (("active break*"[tiab] OR "activity break*"[tiab] OR "movement break*"[tiab] OR "physical activity break*"[tiab] OR "sedentary break*"[tiab] OR "break* up sitting"[tiab] OR "interrupt* sitting"[tiab] OR "sitting interrupt*"[tiab] OR "sedentary interrupt*"[tiab]) AND  ( sedentary[tiab] OR sitting[tiab] OR "sedentary behavio*"[tiab] OR "prolonged sitting"[tiab] OR "sedentary time"[tiab])) | None | 1,175 |
| Scopus (30 Sep 2025) | TITLE-ABS-KEY(( exercise W/1 snack* ) OR "exercise snacking" OR ( movement W/1 snack* ) OR snacktivity OR snacktivity* OR VILPA OR "vigorous intermittent lifestyle physical activity" OR "intermittent lifestyle physical activity") OR TITLE-ABS-KEY(  ((active W/1 break*) OR (activity W/1 break*) OR ( movement W/1 break* ) OR ( physical W/1 activity W/1 break* ) OR ( sedentary W/1 break* ) OR "break up sitting" OR ( break* W/2 sitting ) OR ( interrupt* W/1 sitting ) OR ( sitting W/1 interrupt* ) OR ( sedentary W/1 interrupt* )) AND  (sedentary OR sitting OR ( sedentary W/1 behavio* ) OR "prolonged sitting" OR "sedentary time")) | None | 1,291 |
| Total | (Pre-deduplication) |  | 2466 |

**Notes：**The search strategy was designed to (i) capture the emerging concept and terminology around “exercise snacks” and related constructs (e.g., Snacktivity and vigorous intermittent lifestyle physical activity [VILPA]) and (ii) increase topical specificity for sedentary-behavior interruption by anchoring break/interruption terms to sedentary- and sitting-related keywords.

Reference lists of key reviews and included articles were screened to identify additional relevant studies (reference-list screening/citation tracking).
